# Do job resources buffer the harmful effects of job demands on burnout complaints? A 1-year cohort study of Swedish healthcare professionals

**DOI:** 10.1016/j.ijnsa.2025.100397

**Published:** 2025-08-06

**Authors:** Britta E Gynning, Elin Karlsson, Kevin Teoh, Per Gustavsson, Filip Christiansen, Emma Brulin

**Affiliations:** a Unit of Occupational Medicine, Institute of Environmental Medicine, Karolinska Institutet, Nobels väg 13, 171 65, Solna, Sweden; b Department of Health, Medicine and Caring Sciences, Linköping University, SE-58183, Linköping, Sweden; c The Department of Organizational Psychology, Birkbeck Business School, Birkbeck, University of London, Clore Management Centre, Torrington aquare, Bloomsbury, WC1E 7JL, London, United Kingdom; d Centre for Occupational and Environmental Medicine, Region Stockholm, Solnavägen 4, 113 65, Stockholm, Sweden

**Keywords:** Burnout, professional, Healthcare providers, Health personnel, Longitudinal studies, Moderating variables, Regression analysis, Work environment

## Abstract

**Background:**

The job demands-resources model posits that job resources buffer the effects of job demands on subsequent strain. However, empirical support for this is inconclusive, with some studies suggesting this may be context- or even profession-specific.

**Objective:**

To investigate the buffering effect in the job demands-resources model within the Swedish healthcare sector and the impact of professional differences on this effect

**Method:**

Data were drawn from a 1-year cohort called the *Longitudinal occupational health survey for healthcare in Sweden,* utilising the 2022 and 2023 waves. The study sample consisted of Swedish healthcare professionals who at baseline were 69 years or younger, who participated in both survey waves. In total, the study included 4132 healthcare professionals (1649 physicians, 1631 registered nurses, and 852 nurse assistants). Descriptive statistics and ordinary least squares regression moderation analysis were carried out.

**Results:**

The experience of control at work buffered the impact of several job demands, including quantitative demands (Beta coefficients [*B*] =−0.034, 95 % confidence interval [CI] [−0.05; −0.01]), illegitimate work tasks (*B*=−0.018, 95 % CI [−0.04;−0.01]), effort-reward imbalance (*B* = −0.050, 95 % CI [−0.08; −0.01]), and work-life Interference (*B* = −0.023, 95 % CI [−0.04; −0.004]) on subsequent burnout complaints. Collegial support buffered the effect of emotional demands (*B* = −0.025, 95 % CI [−0.04; −0.01]). Physicians reported a greater buffering effect from control compared with nurse assistants on the effect of illegitimate work tasks (*B* = 0.084, 95 % CI [0.01;0.16]) and effort reward imbalance (*B* = 0.120, 95 % CI [0.02;0.22]) towards subsequent burnout complaints.

**Conclusions:**

We have contributed to the application of the job demands-resources model by emphasising the critical role of the type of profession in the buffering effect of resources. We have underlined the importance of profession-specific job demands and resources in understanding burnout among Swedish healthcare professionals and in other settings.

**What is already known**

• Healthcare professionals in Europe experience some of the highest levels of work-related strain and stress, leading to a heightened risk of becoming burned out.
• The psychosocial work environment differs vastly between, as well as within, distinct healthcare professions.
• Empirical support for the buffer hypothesis, including the buffering effect of resources within the job demands-resources model, is contradictory and inconclusive.

Alt-text: Unlabelled box

**What this paper adds**

• The buffer hypothesis, including the buffering effect of resources proposition within the job demands-resources model, varied by profession, resulting in different patterns of association with burnout complaints.
• Experiencing control at work was the most important job resource to buffer the effect of several job demands on subsequent burnout complaints
• For physicians, the experience of control at work buffered the association between job demands and subsequent burnout complaints more than for nurse assistants also experiencing control at work.

Alt-text: Unlabelled box

## 1 Background

The healthcare sector as a workplace differentiates itself from others by having the survival of its “customers” as its primary foundation (Garman et al., 2006). Simultaneously, the work environment of healthcare professionals is characterised by high job demands with low rewards and few accessible resources, leading to healthcare professionals in Europe experiencing some of the highest levels of work-related strain and stress (European Foundation for the Improvement of Living and Working Conditions., 2016). For healthcare professionals, this strain and stress have been shown to increase the risk of burnout (Brulin et al., 2023; Hagqvist et al., 2022; Kinman et al., 2023; Madsen et al., 2023) and, in turn, sickness absence (Brulin et al., 2025). Understanding the impact of the work environment becomes important to study, not only for the well-being of the healthcare professionals (Christiansen et al., 2024; Manzano-García and Ayala, 2017; Rodrigues et al., 2018) – with corresponding impacts on sickness absence and turnover rates – but also for the safety of their patients, as it may affect job performance (Hall et al., 2016; O’Connor et al., 2020; Salyers et al., 2017; Teoh et al., 2019).

### 1.1 Theoretical framework: The job demand resources model

The job demands-resources model, a widely utilised theoretical framework in occupational health research, suggests that every work environment contains two unique processes, the health impairment process and the motivational process. The health impairment process occurs when elevated job demands result in prolonged effort and subsequent exhaustion. In contrast, the motivational process is initiated by the presence of resources, which enhance motivation and engagement. Job demands are factors of work requiring physical or psychological effort, such as workloads, emotionally exhausting interactions, and administrative obstacles (Demerouti et al., 2001). Long exposure to high demands may lead to burnout; i.e., “a work-related state of exhaustion that occurs among employees, characterised by extreme tiredness, reduced ability to regulate cognitive and emotional processes, and mental distancing” (Schaufeli et al., 2020, p. 4). Conversely, job resources, such as performance feedback, autonomy, and social support, serve two primary functions. They assist employees in achieving their work objectives and promote personal development, while also mitigating the adverse effects of job demands – also called the buffer hypothesis; i.e., the buffering effect of resources (Demerouti et al., 2001; Demerouti and Bakker, 2023). The empirical evidence for the buffer hypothesis is inconclusive (Schaufeli, 2017). Researchers of Dutch higher education employees (Bakker et al., 2005) and researchers of Canadian nurses (Lavoie-Tremblay et al., 2014) found that job resources buffered the association between job demands and strain. Yet, they also argue that the buffer hypothesis is too general; the interaction between job resources and job demands in relation to strain is not always applicable, with much depending on the specific combination of job resources and job demands. Essentially, as described by Fagerlind Ståhl et al. (2018) and others (Häusser et al., 2010; Huth and Chung-Yan, 2023; Teoh and Hassard, 2020), the buffer hypothesis is contextual rather than universal; i.e., the existence of the buffer hypothesis may depend on the research setting, as well as the salience of the job factors being studied. Another factor contributing to inconclusive results may be the neglect of personal resources, as these resources can activate employees' resilience in stressful situations (Xanthopoulou et al., 2007). There are two extensions to the job demands-resources model: the first is personal resources (i.e., individuals' resilience and their capacity to influence and control their environment, which can be demonstrated through traits like optimism and organisational self-esteem [Xanthopoulou et al., 2009]), while the other is engaging leadership (Schaufeli, 2017) (i.e., the leader’s ability to boost employee engagement at both individual and team levels). While the specific role of personal resources in the model remains unclear, it is crucial to recognise personal resources as an integral component of the broader job demand-resource model (Schaufeli, 2017; Xanthopoulou et al., 2007).

The healthcare sector encompasses various care professions, including physicians, registered nurses (RNs), and nurse assistants. Despite this heterogeneity (Garman et al., 2006; Hall, 2005), many studies have treated healthcare professionals as a homogenous group, often overlooking critical professional distinctions (Teoh et al., 2020). However, each professional role presents unique demands and job resources, leading to differing health-related outcomes (Marzocchi et al., 2024; Weeratunga et al., 2024). Not only do these professions differ in responsibilities, economic reimbursement, and promotion possibilities (Barnard et al., 2023), but they also have different professional characteristics relating to values, behaviours, and expectations of their work, derived from their education, as well from the history of their profession (Garman et al., 2006; Hall, 2005). These professional characteristics are also central aspects that may contribute to variations in personal resources (Xanthopoulou et al., 2009). Meanwhile, Xanthopoulou et al. (2009) showed that working environments are antecedents to personal resources, and variations in these environments may also affect variations in personal resources. Indeed, although working in the same setting, previous researchers have shown differences in the psychosocial work environment among healthcare professions in Sweden (Gynning et al., 2024), as well as within the professions themselves, along with varying outcomes (Christiansen et al., 2024; Gynning et al., 2024). This suggests that the differing professional characteristics and working environment associated with the roles of being a physician, RN, or nurse assistant are central in shaping the development and levels of personal resources, which in turn influence their capacity to buffer job demands (Demerouti and Bakker, 2023; Fagerlind Ståhl et al., 2018; Huth and Chung-Yan, 2023; Teoh and Hassard, 2020). Thus, understanding how job resources may buffer the association between job demands and burnout and how professional differences may impact this buffering effect is crucial for future interventions to minimise the risk of burnout among healthcare professionals.

**Fig. 1:**
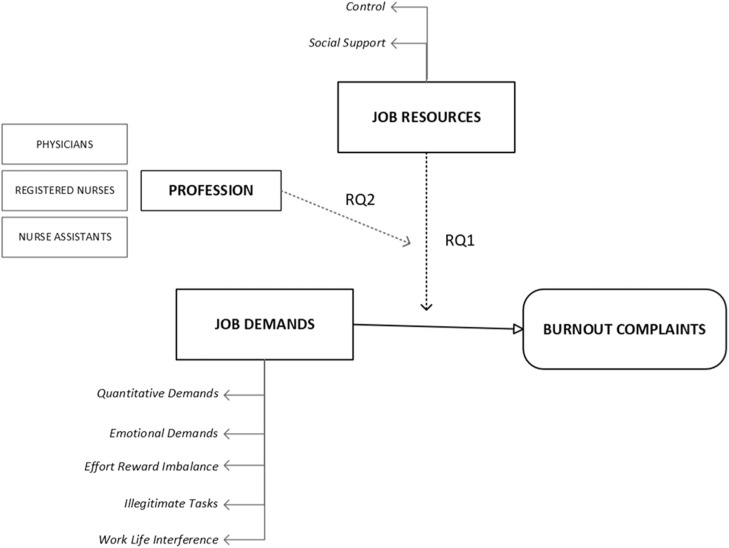
Conceptual framework of the study outlining the theoretical model and corresponding research questions (RQs) guiding the analysis.

Given these complexities, we aimed to investigate the buffering effect in the job demands-resources model within the Swedish healthcare sector and the impact of professional differences on this effect (Fig. 1).

Specifically, we sought to address the following research questions (see the respective research questions in Fig. 1)

1. Do job resources buffer the association between job demands and subsequent burnout complaints, and are there variations in the buffering effect by each healthcare profession?
2. Does the healthcare profession moderate how job resources buffer the association between job demands and subsequent burnout complaints?

## 2 Method

### 2.1 Study setting

This research is based in Sweden, where healthcare is organised under 21 self-governing counties and primarily funded by taxes, ensuring universal access. However, privately funded healthcare services, particularly in primary care, have increased, leading to higher patient costs for some. Social care is managed by self-governing municipalities.

In Sweden, physicians and RNs undergo formal university training, while nurse assistants typically do not have university-level education. Physicians complete an additional 1 year of basic supervised training after certification and thereafter approximately 5 additional years of residency. RNs graduate after 3 years of nursing school and start working immediately.

### 2.2 Data collection and study participation

#### 2.2.1 Data collection

Data for this study originated from the open panel cohort *Longitudinal occupational health survey for healthcare Sweden* (Brulin et al., 2023). This cohort encompassed physicians, RNs, and nurse assistants who had clinically practised in Sweden for the preceding 12 months at recruitment. Participants represented various healthcare settings, including primary care, hospitals, and occupational health services. Some RNs and nurse assistants may have been employed within Swedish municipalities (responsible for health-related social care). Data collection was conducted from March to June of 2022 (baseline) and from October to December 2023 (follow-up).

Baseline data in 2022 included 23,446 healthcare professionals from the Swedish Occupational Register and the Educational Register. The Swedish government agency Statistics Sweden was responsible for the sampling and data collection process. They used a stratified random sampling method, where the strata were six geographical areas, and, for physicians, two additional strata of hospital vs. primary care were used. Potential participants were contacted via postal invitations, allowing them to respond either online or by mail. The invitation encompassed a detailed study description, emphasising the principles of voluntary participation, informed consent, and the participants’ right to withdraw from the study at any point. The total response rate was 32.7 % (*N* = 7658). For each healthcare professional, the survey was distributed to 7908 physicians, of whom 2712 (34.3 %) responded, 7790 RNs, of whom 2903 (37.3 %) responded, and 7748 nurse assistants, of whom 2043 (26.4 %) responded.

The follow-up survey in 2023 was distributed to 24,065 individuals, of whom 6903 (28.7 %) responded. The sample included the baseline cohort and an additional sample of new physicians, RNs, and nurse assistants in the registers since the baseline (*n* = 3364). It excluded individuals who, since baseline, had left the profession, retired, died, or emigrated (*n* = 1310). Per profession, the survey sample of physicians included 7780 individuals, of whom 2867 (36.9 %) responded. For RNs, the 2023 sample included 7634 individuals, of whom 2656 (34.8 %) responded. The 2023 sample for nurse assistants included 7341 individuals, of whom 1380 (18.8 %) responded.

#### 2.2.2 Study participation

In this study, we restricted the sample to those who participated in both data collection phases and were either 69 years or younger at baseline. The total number of individuals who responded to both surveys was 4182. However, with the exclusion of individuals over 69 years of age, the analytical sample of this study consisted of 4132 individuals, of whom 1649 were physicians, 1631 were RNs, and 852 were nurse assistants.

The Swedish Ethical Review Authority approved this study (Review numbers: 2021–05,574–02; 2022–00,310–02; 2022–02,765–02).

#### 2.2.3 Drop-out analysis

Drop-out analysis was performed for all variables (see Supplementary Material Table A). Data collections were analysed for missing data relative to both the analytical sample and the total population, using the Swedish Total Population Register. The missing values analysis compared the characteristics of those participating only in the baseline collection with those participating in both baseline and follow-up.

The drop-out analysis revealed that 58 % of women and 54 % of men participated at both time points. Among professions, 62.9 % of female and 62.3 % of male physicians, 56.6 % of female and 52.4 % of male RNs, and 42.6 % of female and 31.1 % of male nurse assistants participated at both time points. The prevalence of burnout risk was 8.3 % among those responding only in 2022 and 5.5 % for those answering both 2022 and 2023. The largest difference among professions was seen in RNs, with a prevalence of 7.0 % for those responding only in 2022, compared to 4.3 % for those responding in both 2022 and 2023.

### 2.3 Study measures

We employed various measures of job demands and job resources, selected based on previous studies where these specific measurements were linked to having a substantial role in the working life of Swedish healthcare workers (Brulin and Elsert Gynning, 2023; Gynning et al., 2024). Thus, these measurements were deemed most suitable due to their proven relevance and applicability in similar contexts. Data on each job demand, job resource, and potential confounder were drawn from the 2022 baseline, while data concerning burnout complaints were utilised from both the 2022 baseline and 2023 follow-up. The Cronbach’s alpha (α) for the total sample for each study measure is presented below. Each measurement item, scale and α for each profession can be found in the Supplementary Material (Supplementary Material Table B).

#### 2.3.1 Outcome: burnout complaints

*Burnout complaints* were assessed using the Burnout Assessment Tool (Schaufeli et al., 2020), translated to Swedish and validated (Hadžibajramović et al., 2022). The Burnout Assessment Tool aligns with the job demands-resources model, indicating that burnout is positively related to job demands and negatively to resources (De Beer et al., 2022). The short version, called BAT12 (Hadžibajramović et al., 2020), includes 12 items on a 5-point Likert scale (1: "Never" to 5: "Always"). Burnout complaints were calculated as grand means for each year (Baseline α: 0.901 & Follow-up α: 0.906), with higher scores denoting greater complaints.

The BAT12 was dichotomised for drop-out analysis at a cutoff of 2.96, indicating burnout risk (Schaufeli and De Witte, 2023).

#### 2.3.2 Exposures

##### 2.3.2.1 Job demands

*Quantitative- and Emotional Demands:* Quantitative demands were assed with three items (5-point Likert scale; α: 0.874). Emotional demands were measured using one item. Higher scores indicate greater demands. All of the items derive from the Swedish version of the Copenhagen Psychosocial Questionnaire (Berthelsen et al., 2020; Burr et al., 2019).

To measure *Illegitimate Work Tasks*, the Bern Illegitimate Task Scale (Jacobshagen, 2006) translated to Swedish, was used (Stengård et al., 2024). The scale has eight items measuring unnecessary and unreasonable tasks, combined into a grand mean score (α: 0.855), with a higher number indicating more illegitimate tasks.

*Effort-Reward Imbalance*, based on the questionnaire by Siegrist et al, (2004) was translated and validated in Sweden (Leineweber et al., 2010). The questionnaire encompasses three items measuring effort (α: 0.784) and seven items measuring rewards (α: 0.773), and each item uses a 4-point Likert scale. As described by Sigrist et al, (2004), a ratio of work effort to rewards was calculated by dividing the effort score by the reward score and applying a correction factor (c = effort/reward = 3/7). A ratio greater than 1 indicates an imbalance, with more effort than reward.

*Work-Life Interference* was assessed using a Swedish translation of the subset of the scale proposed by Fisher and colleagues (Fisher et al., 2009; Magnusson Hanson et al., 2018). The scale assesses the imbalance between work and private life using five items on a 5-point Likert scale. The grand mean scores range from 1 to 5 (α: 0.929), with higher scores indicating greater Work Life Interference.

##### 2.3.2.2 Job resources

*Control was* measured through items adapted from the Swedish Longitudinal Occupational Survey of Health (Albrecht et al., 2017; Magnusson Hanson et al., 2018). Control was assessed through 10 items (6-point Likert scale), including items concerning control over work content, over work time, and the level of influence at work. The 10 items were compiled into a grand mean score ranging from 1–5 (α: 0.892). Responses marked “Not relevant” (6th option) were treated as missing, and the grand mean followed the ‘half-rule’ (Bell et al., 2016).

*Social support,* derived from the Swedish translation of the Copenhagen Psychosocial Questionnaire (Berthelsen et al., 2020; Burr et al., 2019), was measured using two separate items: (1) collegial and (2) managerial. Both had answers on a 5-point Likert scale. Items referred to the availability of help and support at work from either colleagues or their first-line manager.

#### 2.3.3 Potential confounders

The analysis considered several potentially-confounding variables chosen and derived from previous literature (Larsen et al., 2020; McVicar, 2016) and their potential impact on both job demands and resources, as well as burnout complaints. Confounders were collected from the baseline data. Confounders included *age* treated as a continuous variable, *sex* classified as (1) men or (2) women, and *birth* country classified as (1) Sweden-born or (2) foreign-born. Further confounders included *years of working experience,* categorised into (1) < 5 years, (2) 5–15 years, and (3) >15 years, and *self-estimated number of working hours,* categorised as (1) <36 hours of work per week, (2) 36–40 h/week, and (3) >40 h/week.

The healthcare *workers’ place*- and *county of work,* were also included to adjust for skewed sampling during data collection and implemented to mitigate selection bias. *Workplace,* categorised into mainly working in (1) primary care, (2) municipality, (3) hospital, or (4) other (occupational health service, consulting, or other). The *County of work* represented the 21 self-governing counties of Sweden and was kept as a multiple-categorical variable.

### 2.4 Statistical analysis

All analyses were conducted using IBM SPSS Statistics version 28.0.

#### 2.4.1 Descriptive statistics

Descriptive statistics using frequencies were computed to describe the analytical sample. Mean tables were computed to determine the total means regarding burnout complaints, job demands, and job resources. Pearson correlation was tested to review the correlation between each measure.

#### 2.4.2 Moderation analysis

To address research questions 1 and 2, ordinary least squares regression moderation analyses were conducted using PROCESS Hayes Macro (Hayes, 2022).

For research question 1, we employed PROCESS analytical moderation model 1 (Hayes, 2022) to investigate the buffering effect of job resources on the association between job demands and burnout complaints (Y = Burnout complaints; X = Job demands; and M [moderator] = Job resources; see Fig. 1). Next, we stratified the results by conducting separate analyses for each profession.

For research question 2, we employed PROCESS analytical moderation model 3 (Hayes, 2022) to test the healthcare profession as a second-order moderator (i.e., effect modification) on the interaction between job demands and job resources, comparing the buffering effect across professions (see Fig. 1). Physicians were implemented as the reference category. Sensitivity analyses were also conducted for PROCESS model 3 using nurse assistants as the reference category, though no statistically significant moderation was found.

Each PROCESS model was controlled for baseline burnout complaints, run with a bootstrap of 15.000. Results from the moderation analyses are presented as unstandardized beta coefficients (*B*) with 95 % confidence intervals (95 % CI).

To explore the moderation analysis further, we plotted the statistically significant moderation associations, including the -1 Standard Deviation (SD), mean, and +1 SD for the statistically significant moderating job resources.

## 3 Results

### 3.1 Description of study sample

Table 1 describes the study sample. The study sample was predominantly female, with notable sex differences across professions. Nurse assistants were generally older than other groups, while physicians were the youngest. Most participants had extensive work experience and were born in Sweden. A considerable number reported working beyond the Swedish standard of 40 hours per week, particularly physicians. Workplace setting also varied by profession, with physicians and RNs primarily based in hospitals, while nurse assistants were more often employed in municipalities, such as in elderly care facilities.

**Table 1:** Demographic description and work factors divided by the total and healthcare profession (baseline 2022).

	Total		Physicians	RNs^a^	Nurse Asst.^b^
	***N* (%)**		***n* (%)**	***n* (%)**	***n* (%)**
**Total**	4132(100)		1649(39.9)	1631(39.5)	852(20.6)
**Demographics**					
*Sex*					
Men	890(21.5)		689(41.8)	151(9.3)	50(5.9)
Women	3242(78.5)		960(58.2)	1480(90.7)	802(94.1)
*Age*					
Mean age (Standard Deviation)		47.5 (11.8)	44.3(11.4)	48.0(12.1)	52.6(10.1)
*Birth Country*					
Sweden-born	3604(87.6 %)		1366(83.3 %)	1498(92.2 %)	740(86.9 %)
Foreign-born	512(12.4 %)		273(16.7 %)	127(7.8 %)	112(13.1 %)
Missing	16(0.4)		10(0.6)	6(0.4)	0(0)
**Work factors**					
*Years of working experience*					
<5 years	559(13.6)		300(18.2)	213(13.1)	46(5.4)
5–15 years	1394(33.8)		675(41.0)	485(29.8)	234(27.6)
>15 years	2172(52.7)		672(40.8)	932(57.2)	568(67.0)
Missing	7(0.2)		2(0.1)	1(0.1)	4(0.5)
*Self-estimated number of working hours per week*					
<36h	1119(27.2)		233(14.2)	500(30.8)	386(45.8)
36–40h	1251(30.4)		314(19.1)	602(37.0)	335(39.7)
>40h	1743(42.4)		1097(66.7)	524(32.2)	122(14.5)
Missing	19(0.5)		5(0.3)	5(0.3)	9(1.1)
*Workplace*					
Primary care	841(20.4)		576(35.0)	234(14.4)	31(3.7)
Municipality	804(19.5)		N/A	255(15.6)	549(64.9)
Hospital	1935(46.9)		913(55.4)	816(50.1)	206(24.3)
Other	544(13.2)		159(9.6)	325(19.9)	60(7.1)
Missing	8(0.2)		1(0.1)	1(0.1)	6(0.7)

a Registered Nurses,

b Nurse Assistants

#### 3.1.1 Mean value patterns in work factors and burnout complaints

Results for the mean values and correlation for each measurement can be found in the Supplementary Material, Table C. Burnout complaints showed a slight overall decline from 2022 to 2023 (mean 2022 1.86, 95 % CI [1.84; 1.88]); mean 2023 1.82, 95 % CI [1.80; 1.84]). While physicians initially reported the highest burnout complaint levels in 2022 (mean 1.81, 95 % CI [1.78; 1.84]), nurse assistants surpassed them in 2023 (mean 1.87, 95 % CI [1.82; 1.91]).

Across professions, physicians experienced the highest quantitative demands (mean 3.24, 95 % CI [3.19; 3.28]), illegitimate work tasks (mean 3.08, 95 % CI [3.05; 3.12]), and work-life interference (mean 3.15, 95 % CI [3.10; 3.20]). RNs reported the highest emotional demands (mean 3.40, 95 % CI [3.35–3.46]), and nurse assistants faced the greatest effort-reward imbalance (mean 1.45, 95 % CI [1.40; 1.49]). However, physicians also reported the highest mean values of managerial support (mean 3.68, 95 % CI [3.62; 3.73]), while RNs experienced the strongest collegial support (mean 1.45, 95 % CI [1.40; 1.49]) and control at work (mean 2.87, 95 % CI [2.82; 2.91]).

### 3.2 Do job resources buffer the association between job demands and subsequent burnout complaints?

Table 2 outlines the findings for research question 1. Positive coefficient values (*B*) indicate a one-unit increase in burnout complaints, while negative values suggest a one-unit decrease.

**Table 2:** Moderation analysis of the buffering effect from job resources on the association between job demands and subsequent burnout complaints, for the entire sample and stratified by profession1.

		**Total sample (*N*=3496)**		**Stratified by profession**					
				**Physicians (*n*= 1424)**		**RNs**^a^**(*n*=1405)**		**Nurse Asst**^b^**(*n*=667)**	
		**Direct effect**	**Moderating effect**	**Direct effect**	**Moderating effect**	**Direct effect**	**Moderating effect**	**Direct effect**	**Moderating effect**
		Demand → Burnout complaints	Resource ↓ Demand → Burnout complaints	Demand → Burnout complaints	Resource ↓ Demand → Burnout complaints	Demand → Burnout complaints	Resource ↓ Demand → Burnout complaints	Demand → Burnout complaints	Resource ↓ Demand → Burnout complaints
		*B* (95 % CI)	*B* (95 % CI)	*B* (95 % CI)	*B* (95 % CI)	*B* (95 % CI)	*B* (95 % CI)	*B* (95 % CI)	*B* (95 % CI)
Control	Emotional Demands	−0.023(−0.07;0.03)	0.005(−0.01;0.02)	−0.053(−0.13;0.03)	0.009(−0.02;0.04)	−0.008(−0.09;0.07)	0.007(−0.02;0.03)	−0.004(−0.12;0.11)	−0.002(−0.04;0.04)
	Quantitative Demands	0.148(0.08;0.21)*	−0**.034(**−0**.05;**−0.01)*	0.211(0.11;0.31)*	−0**.053(**−0**.08;**−0.02)*	0.070(−0.03;0.17)	−0.013(−0.05;0.02)	0.100(−0.05;0.25)	−0.018(−0.07;0.04)
	Illegitimate Work Tasks	0.148(0.08;0.21)*	−0**.018(**−0**.04;**−0.01)*	0.280(0.15;0.41)*	−0**.052(**−0**.10;**−0.01)*	0.110(−0.01;0.24)	−0.006(−0.04;0.03)	−0.044(−0.22;0.13)	0.040(−0.02;0.10)
	Effort Reward Imbalance	0.160(0.07;0.25)*	−0**.050(**−0**.08;**−0.01)*	0.301(0.13;0.47)*	−0**.101(**−0**.17;**−0.04) *	0.109(−0.04;0.26)	−0.04(−0.09;0.02)	0.048(−0.14;0.23)	0.033(−0.04;0.11)
	Work-Life Interference	0.264(0.21;0.32)*	−0**.023(**−0**.4;**−0.004)*	0.252(0.16;0.35)*	−0.014(−0.05;0.02)	0.259(0.17;0.35)*	−0.026(−0.05;0.002)	0.241(0.11;0.37)*	−0.012(−0.06;0.04)
Manager Support	Emotional Demands	0.011(−0.04;0.06)	−0.005(−0.02;0.01)	0.016(−0.06;0.09)	−0.012(−0.03;0.01)	0.024(−0.05;0.10)	−0.003(−0.02;0.02)	−0.028(−0.15;0.09)	0.005(−0.03;0.04)
	Quantitative Demands	0.083(0.02;0.14)*	−0.009(−0.02;0.01)	0.092(−0.01;0.19)	−0.009(−0.03;0.02)	0.050(−0.04;0.14)	−0.005(−0.03;0.02)	0.066(−0.09;0.22)	−0.003(−0.04;0.04)
	Illegitimate Work Tasks	0.151(0.07;0.23)*	−0.01(−0.03;0.01)	0.217(0.08;0.35)*	−0.020(−0.05;0.01)	0.141(0.02;0.26)*	−0.014(−0.04;0.02)	0.030(−0.014;0.20)	0.009(−0.03;0.05)
	Effort Reward Imbalance	0.045(−0.04;0.13)	0.001(−0.02;0.02)	0.042(−0.10;0.19)	0.010(−0.03;0.05)	0.041(−0.09;0.17)	−0.008(−0.05;0.03)	0.091(−0.08;0.27)	0.008(−0.04;0.06)
	Work-Life Interference	0.250(0.19;0.30)*	−0.014(−0.083;0.0003)	0.271(0.18;0.36)*	−0.016(−0.04;0.01)	0.248(0.16;0.33)*	−0.018(−0.04;0.004)	0.206(0.09;0.32)*	0.001(−0.03;0.03)
Collegial Support	Emotional Demands	0.100(0.01;0.19)*	−0**.025(**−0**.04;**−0.01)*	0.030(−0.09;0.15)	−0.013-(0.04;0.01)	0.159(0.004;0.31)*	−0.033(−0.07;0.002)	0.167(−0.07;0.40)	−0.040(−0.09;0.01)
	Quantitative Demands	0.002(−0.10;0.11)	0.011(−0.01;0.03)	−0.038(−0.20;0.12)	0.022(−0.01;0.06)	−0.003(−0.19;0.18)	0.008(−0.03;0.05)	−0.079(−0.36;0.20)	0.031(−0.03;0.09)
	Illegitimate Work Tasks	0.020(−0.12;0.16)	0.019(−0.011;0.05)	0.067(−0.14;0.27)	0.017(−0.03;0.06)	−0.098(−0.34;0.14)	0.04(−0.01;0.09)	−0.041(−0.36;0.28)	0.023(−0.05:0.09)
	Effort Reward Imbalance	−0.041(−0.19;0.11)	0.021(−0.01;0.06)	−0.073(−0.29;0.14)	0.036(−0.01;0.09)	0.031(−0.24;0.30)	−0.003(−0.06;0.06)	−0.111(−0.48;0.26)	0.053(−0.03;0.14)
	Work-Life Interference	0.259(0.16;0.36)*	−0.013(−0.03;0.01)	0.209(0.06;0.36)*	0.001(−0.03;0.03)	0.360(0.20;0.52)*	−0**.039(**−0**.07;**−0.004)*	0.141(−0.09;0.37)	0.016(−0.04;0.07)

⁎ P-value <0.05

1 Adjusted for sex, age, years of working experience, working hours per week, workplace & county of work (& profession for the total sample)

a Registered Nurses

b Nurse Assistants N = The total number of observations in the study sample n = The number of observations in each healthcare professional group of the total sample *B* = Unstandardized beta coefficients 95 % CI = 95 % confidence interval

As shown in the second column of Table 2, we found that certain job resources – particularly control and collegial support – significantly buffered the impact of job demands on burnout complaints. Specifically, control buffered the effects of quantitative demands, illegitimate work tasks, effort reward imbalance, and work-life interference. Collegial support specifically buffered the association between emotional demands and burnout complaints.

**Fig. 2:**
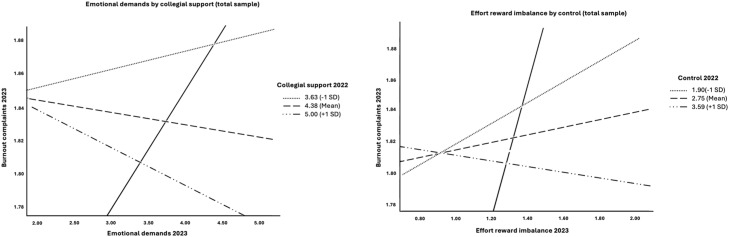
Emotional demands by collegial support and effort reward imbalance by control, entire study sample. The legend represents the coefficients for each level of moderation, showing -1 standard deviation from the mean (-1SD), the mean value, and +1 standard deviation from the mean (+1 SD). The figure indicates that for each demand (emotional demands and effort rewards imbalance), high moderation from the respective resource (+1SD) appeared to weaken and even reverse the impact of the job demands on burnout complaints. Filled line = reference line between emotional demands and subsequent burnout complaints without a moderating effect. Note that the Y-axis is broken.

Fig. 2 shows that high job resources (+1 SD from the mean) weakened or even reversed the impact of emotional demands (moderated by collegial support) and effort rewards imbalance (moderated by control). See Figures A, B, and C in the supplementary material for further significant plots.

#### 3.2.1 Are there variations by each profession?

The last columns of Table 2 display the stratified results of research question 1. Physicians appeared to benefit most from job control, which significantly buffered the impact of several demands. For RNs, collegial support was particularly effective in buffering work–life interference. In contrast, no statistically significant buffering effect was observed among nurse assistants.

Plots for the stratified sample showed that for physicians, high control negatively affected the association between effort reward imbalance and burnout complaints (see Supplementary Material Figure F).

#### 3.2.2 Does the healthcare profession moderate how job resources buffer the association between job demands and subsequent burnout complaints?

Table 3 outlines the findings for research question 2. Columns A and B, shown under the column “Moderating effect”, display a second-order moderation (i.e., effect modification) of the profession on the buffering effect, with physicians as the reference group. Column B indicates that control had a stronger protective effect for physicians compared to nurse assistants, particularly in relation to illegitimate work tasks and effort-reward imbalance. See supplementary material Figures H & I for comparing plots.

**Table 3:** Moderation analysis for the buffering effect of job resources with the moderating effect of the healthcare profession (*N* = 3496)1.

		**Direct effect**	**Moderating effect**		
		**Demand → Burnout complaints**	**Resource** **↓** **Demand → Burnout complaints**	**Column A**	**Column B**
				**Resource** **RNs**^2^^,^^a^^.^**→↓** **Demand → Burnout complaints**	**Resource** **Nurse Asst.**^2^^,^^b^^.^**→↓** **Demand → Burnout complaints**
		*B* (95 % CI)	*B* (95 % CI)	*B* (95 % CI)	*B* (95 % CI)
**Control**	**Emotional Demands**	−0.042(−0.12;0.04)	0.009(−0.02;0.04)	−0.0003(−0.04;0.04)	−0.015(−0.06;0.03)
	**Quantitative Demands**	0.231(0.13;0.33)*	−0**.058(**−0**.09;**−0.02)*	0.046(−0.001;0.09)	0.031(−0.03;0.09)
	**Illegitimate Work Tasks**	0.293(0.16;0.42)*	−0**.056(**−0**.10;**−0.01)*	0.051(−0.01;0.11)	0.084(0.01;0.16)*
	**Effort Reward Imbalance**	0.327(0.16;0.49)*	−0**.097(**−0**.16;**−0.03)*	0.057(−0.03;0.14)	0.120(0.02;0.22)*
	**Work-Life Interference**	0.266(0.17;0.36)*	−0.02(−0.05;0.01)	−0.005(−0.05;0.04)	−0.004(−0.06;0.05)
**Managerial Support**	**Emotional Demands**	0.022(−0.05;0.10)	−0.011(−0.03;0.01)	0.009(−0.02;0.04)	0.014(−0.02;0.05)
	**Quantitative Demands**	0.092(−0.01;0.19)	−0.010(−0.03;0.02)	0.002(−0.04;0.04)	0.003(−0.04;0.05)
	**Illegitimate Work Tasks**	0.221(0.09;0.35)*	−0.022(−0.06;0.01)	0.011(−0.03;0.06)	0.029(−0.02;0.08)
	**Effort Reward Imbalance**	0.055(−0.09;0.20)	0.011(−0.03;0.05)	−0.022(−0.08;0.03)	−0.008(−0.07;0.05)
	**Work-Life Interference**	0.275(0.18;0.36)*	−0.016(−0.04;0.01)	−0.001(−0.03;0.03)	0.017(−0.02;0.05)
**Collegial Support**	**Emotional Demands**	0.056(−0.07;0.18)	−0.017(−0.05;0.01)	−0.016(−0.06;0.03)	−0.022(−0.08;0.03)
	**Quantitative Demands**	−0.034(−0.20;0.13)	0.023(−0.01;0.06)	−0.019(−0.07;0.04)	0.002(−0.07;0.07)
	**Illegitimate Work Tasks**	0.077(−0.13;0.28)	0.016(−0.03;0.06)	0.025(−0.04;0.09)	0.008(−0.07;0.09)
	**Effort Reward Imbalance**	−0.066(−0.28;0.15)	0.041(−0.01;0.09)	−0.046(−0.13;0.03)	0.011(−0.08;0.10)
	**Work-Life Interference**	0.217(0.07;0.37)*	0.001(−0.03;0.03)	−0.038(−0.09;0.01)	0.013(−0.05;0.07)

⁎ P-value <0.05

1 Adjusted for sex, age, years of working experience, working hours per week, workplace & county of work

2 Reference category physicians

a Registered Nurses

b Nurse Assistants N = The total number of observations in the study sample *B* = Unstandardized beta coefficient 95 % CI = 95 % confidence interval

No statistical differences were observed comparing physicians and RNs (Column A).

## 4 Discussion

In this study, we investigated the buffer hypothesis, specifically the buffering effect within the job demands-resources model in the Swedish healthcare sector, focusing on the role of different professions.

Our 1-year follow-up found that control buffered several demands on the healthcare professional workforce, while collegial and managerial support did less so. Specifically, control mitigated the negative effects of quantitative demands, illegitimate work tasks, effort-reward imbalance, and work-life interference on burnout (research question 1). Among healthcare professionals, only physicians experienced a buffering effect from control for quantitative demands, illegitimate work tasks, and effort-reward imbalance. RNs showed a buffering effect only for work-life interference facilitated by collegial support (research question 1). When comparing professions having access to job resources, physicians demonstrated a more significant buffering effect from control than nurse assistants regarding illegitimate work tasks and effort-reward imbalance (research question 2).

Unlike Fagerlind Ståhl et al. (2018), who rejected the buffer hypothesis with the buffering effect of the job demands-resources model, we found a profession-dependent buffering effect related to professional differences, aligning with the suggestion by Bakker et al. (2005). We have furthered the knowledge of previous research, emphasising the need for profession-specific perspectives within healthcare research (Madsen et al., 2023), particularly in applying the job demands-resources model (Huth and Chung-Yan, 2023; Marzocchi et al., 2024; Teoh and Hassard, 2020; Weeratunga et al., 2024).

Menghini and Balducci (2021) highlighted the need for contextualised indicators to measure workplace stress in the Italian healthcare sector, cautioning against standardised measurements that may overlook the complexities of organisational structures affecting well-being. We agree with this statement, as we found that varying levels of control related to job demands could have either no buffering effect or a reverse negative association with burnout complaints, depending on profession. One explanation for variations in the buffering effects between healthcare professions may be attributed to their professional characteristics and work environments, which shape their personal resources. Although we did not measure individual personal resources, these professional differences may act as a useful proxy. According to Xanthopoulou et al. (2009), the work environment significantly influences personal resources. Physicians, RNs, and nurse assistants work in diverse environments that are impacted by different conditions and demands (Gynning et al., 2024). For example, Christiansen et al. (2024) reported large variations in the prevalence of effort-reward imbalance and subsequent risk of burnout across physician specialities. Similarly, Ahlstedt et al. (2024) found that the association between work motivation, as well as the type of support, varied among nurse assistants who worked in hospitals, primary care, and home healthcare. Therefore, the variations in how certain resources buffer the relationship between demands and burnout can be partially explained by these professional differences, varying work environments, and, ultimately, the personal resources that affect individuals' ability to utilize available job resources effectively.

Differences in the buffering effects among professions may also stem from the nature of the job demands and resources measured. As Häusser et al. (2010), Lavoie-Tremblay et al. (2014), and Fagerlind Ståhl et al. (2018) note, the latent dimensions of job resources and job demands significantly influence their interaction towards burnout outcomes; i.e., social support is related to the feeling of being supported and cared for, while control relates to the feeling of having structure and influence. Thus, each job resource may naturally buffer only job demands measuring similar dimensions and aspects of work (Fagerlind Ståhl et al., 2018), such as emotional- or quantitative demands. Karasek’s (1979) demand-control-support theory posits similar ideas describing how different job resources relate to different capabilities. Job control relates to the extent to which an individual can manage work tasks, while social support involves helpful and available social interaction (Karasek and Theorell, 1990). In our study, these patterns were observed: control mitigated the effects of workload-related demands, while collegial support buffered emotional demands. However, for nurse assistants, no statistically significant buffering effects were found. One plausible explanation is that nurse assistants perform distinct tasks and are exposed to different job demands and resources compared to other healthcare professionals. Thus, their work environment – often situated in municipal elderly care – may not align with the demand-resource integration found among physicians and RNs. As such, the moderating effect of job control or social support may be less relevant or different among nurse assistants. These differences underscore the importance of considering professional differences when applying stress and coping models in healthcare settings, as different job resources may target distinct aspects of work and prove effective in different situations.

A way forward in applying the buffer hypothesis within the job demands-resources model is to implement it as a framework and an organising structure rather than as a strictly testable hypothesis. As suggested by Huth and Chung-Yan (2023), this approach emphasises the need to adapt theories and models to specific contexts to understand the interplay between individuals and their environment. We can, in that way, provide a theoretical contribution to the job demands-resources model that may become useful for other researchers studying the healthcare sector and how components of the model might apply differently to various professions. Regarding our study, it is essential to recognise the healthcare settings as heterogeneous organisations characterised by diverse professional roles and vulnerable time windows. This understanding is vital for comprehending the well-being of healthcare professionals, as each profession is shaped by different professional characteristics, including distinct values, cultures, expectations, as well as personal resources influenced by education and the profession’s historical context (Garman et al., 2006; Hall, 2005).

Ultimately, understanding the unique demands and resources available to different healthcare professions is critical in addressing burnout and promoting well-being. The insights gained from this study emphasise the importance of professional differences in shaping responses to job demands. By focusing on profession-specific characteristics and implementing supportive structures tailored to those characteristics, healthcare organisations can better equip their workforce to handle challenges effectively. This ultimately may lead to a more resilient healthcare system, better patient care, and improved overall employee satisfaction.

### 4.1 Practical implications

Given the critical role of the work environment in healthcare professionals' retention and well-being, we have highlighted the need to address specific job demands to improve working conditions across distinct professional groups. Reducing excessive job demands should be a key priority, especially considering the limited and uncertain buffering effects of job control. While previous researchers (Schaufeli, 2017) have shown that job resources can help prevent burnout and promote engagement, minimising job demands remains essential for effectively reducing burnout once it has occurred.

Future researchers should focus on developing and evaluating tailored interventions that address the unique job demands and resources of specific healthcare professions. This includes both qualitative studies to understand profession-specific needs and experimental or longitudinal studies to assess the effectiveness of targeted strategies. We have also highlighted the need for appropriate risk assessments and staff participation in interventions to understand specific local demands and resources, including how they present for different professions. For example, interventions for physicians may focus on reducing administrative burdens through increased control, while for RNs, increased support from colleagues may help mitigate work-life conflict. Understanding these nuances is key to designing effective, sustainable interventions to reduce burnout and enhance staff retention.

### 4.2 Strengths and limitations

This study has notable strengths and limitations. Its two time points allowed for causal testing between job demands and burnout complaints, addressing criticism of previous research with poor methodological quality (Lesener et al., 2019) or cross-sectional design (Marzocchi et al., 2024). Additionally, by differentiating among three healthcare professions, we have provided valuable comparisons and highlighted the heterogeneity often overlooked in healthcare research. The large and fairly representative sample allowed us to draw conclusions beyond the Swedish context, emphasising the importance of other studies to consider the local perspectives of professions.

However, there are limitations to consider, such as attrition between 2022 and 2023. In the drop-out analysis, burnout complaint levels in 2022 were lower among respondents who answered both surveys. This may suggest a healthy worker effect, indicating that those with higher burnout complaints might have been less likely to respond. This should be considered when interpreting results, as actual burnout levels could be higher.

Another limitation is using single items, specifically emotional demands and social support from managers and colleagues. This limited the psychometric validity of the measures, as they may not represent the complexities of the phenomenon being measured. Moreover, conceptually, there are other aspects of the job demands-resources model not considered in this study, specifically concerning other types of job resources. For example, within the healthcare sector, other important job resources concern rewards, including prospects for professional growth, organisational fairness, and staff adequacy (Marzocchi et al., 2024). However, more recent iterations of the job demands-resources model focus on the role of personal resources, such as resilience and self-efficacy and engaging leadership, both of which may play an important part in the way job resources are allocated. This warrants future studies, as these resources can influence workplace dynamics and well-being.

## 5 Conclusions

We have advanced the job demands-resources model by highlighting the role of the profession as a moderating factor for the buffering of resources in the demand-burnout complaint association in the Swedish healthcare sector. We have shown that job resources, especially control and social support, buffer the effects of specific job demands differently depending on the profession. This underscores the significance of profession-specific job demands and resources in understanding burnout among Swedish healthcare professionals and potentially those in other countries.

## Declarations

### Consent for publication

All survey participants consented to publication when they logged in to the web survey or posted the paper survey.

### Prior use of dataset

This work is part of a larger cohort, Longitudinal occupational health survey for healthcare Sweden (LOHHCS), and has similarities with previously published work utilising the full sample (physicians, RNs, and nurse assistants), as well as similar variables; e.g., the Burnout Assessment Tool, quantitative demands, and work-life interference. Previous researchers have focused on either one specific profession, one specific theoretical model, or cross-sectional data. In this paper, we investigated the buffering effect within the job demands-resources model and compared healthcare professions, for which these data have not been utilised before.

Similar studies include: Gynning, Britta Elsert, Elin Karlsson, Kevin Teoh, Per Gustavsson, Filip Christiansen, and Emma Brulin. ”Contextualising the Job Demands–Resources Model: A Cross-Sectional Study of the Psychosocial Work Environment across Different Healthcare Professions”. *Human Resources for Health* 22, nr 77 (2024).

## Funding

This work was supported by the Swedish Research Council (grant number: 2022–00,806) and followed the Strengthening the Reporting of Observational Studies in Epidemiology (STROBE) reporting guidelines for cohort studies (von Elm et al., 2008).

## CRediT authorship contribution statement

**Britta E Gynning:** Writing – original draft, Methodology, Investigation, Formal analysis, Conceptualization. **Elin Karlsson:** Writing – review & editing, Supervision, Conceptualization. **Kevin Teoh:** Writing – review & editing, Supervision, Methodology, Formal analysis, Conceptualization. **Per Gustavsson:** Writing – review & editing, Supervision. **Filip Christiansen:** Writing – review & editing. **Emma Brulin:** Writing – review & editing, Supervision, Project administration, Methodology, Investigation, Funding acquisition, Formal analysis, Conceptualization.

## Declaration of competing interest

The authors have nothing to declare.

## Appendix Supplementary materials

Image, application 1
