# Supplementary material for: Do job resources buffer the harmful effects of job demands on burnout complaints? A 1-year cohort study of Swedish healthcare professionals
Source: Int J Nurs Stud Adv. 2025 Aug 6;9:100397. doi: 10.1016/j.ijnsa.2025.100397 (PMC12359236; doi:10.1016/j.ijnsa.2025.100397)
Supplement: Supplementary file 1 [file mmc1.docx]

*Do job resources buffer the harmful effects of job demands on burnout complaints? A 1-year cohort study of Swedish healthcare professionals.*

Supplementary material

Supplementary Table A: Drop-out analysis

| **Supplementary Table A: Drop-out analysis** | | | | | | | | | | | | |
| --- | --- | --- | --- | --- | --- | --- | --- | --- | --- | --- | --- | --- |
|  | *Only respondents Baseline 2022* | | | | *Respondents Baseline 2022 and Follow-up 2023* | | | | *Percentage within category responding twice* | | | |
|  | *Total* | *Physician* | *Registered nurses* | *Nurse Assistants* | *Total* | *Physician* | *Registered nurses* | *Nurse Assistants* | *Total* | *Physician* | *Registered nurses* | *Nurse Assistants* |
| **Sex** | | | | | | | | | | | | |
| Women | 80.2 | 56.7 | 89.2 | 90.7 | 77.8 | 57.2 | 90.7 | 94.1 | 57.5 | 62.9 | 56.6 | 42.6 |
| Men | 19.8 | 43.3 | 10.8 | 9.3 | 22.2 | 42.8 | 9.3 | 5.9 | 53.8 | 62.3 | 52.4 | 31.1 |
| **Age** | | | | | | | | | | | | |
| >37 | 29.0 | 35.3 | 34.5 | 17.8 | 24.7 | 31.9 | 23.7 | 12.3 | 50.6 | 60.2 | 46.9 | 33.1 |
| 38-48 | 23.8 | 30.3 | 23.1 | 19.1 | 26.3 | 32.8 | 25.3 | 15.3 | 57.1 | 64.5 | 58.4 | 36.4 |
| 49-58 | 23.9 | 16.2 | 21.2 | 33.2 | 25.0 | 17.0 | 26.9 | 37.3 | 55.8 | 63.8 | 61.9 | 44.5 |
| 58+ | 23.3 | 18.1 | 21.1 | 29.9 | 24.0 | 18.2 | 24.1 | 35.1 | 55.3 | 62.8 | 59.4 | 45.6 |
| **Birth Country** | | | | | | | | | | | | |
| Within Sweden | 82.6 | 76.6 | 90.9 | 78.9 | 87.7 | 83.7 | 92.2 | 86.9 | 56.2 | 64.8 | 56.7 | 44.0 |
| Outside of Sweden | 17.4 | 23.4 | 9.2 | 21.1 | 12.4 | 16.3 | 7.8 | 13.2 | 46.1 | 53.9 | 52.2 | 30.9 |
| **Years of working experience** | | | | | | | | | | | | |
| <5 years | 17.4 | 24.5 | 20.6 | 7.8 | 13.4 | 17.7 | 13.1 | 5.4 | 48.2 | 54.8 | 44.9 | 33.3 |
| 5-15 years | 32.4 | 36.6 | 30.4 | 31.1 | 33.4 | 39.8 | 29.8 | 27.6 | 55.4 | 64.7 | 55.8 | 38.9 |
| >15 years | 50.2 | 38.9 | 49.0 | 61.1 | 53.2 | 42.5 | 57.2 | 67.0 | 56.2 | 64.8 | 60.1 | 44.0 |
| **Working Hours** | | | | | | | | | | | | |
| Under 36h/w | 28.2 | 15.2 | 30.3 | 37.1 | 27.8 | 15.9 | 30.8 | 45.8 | 54.3 | 63.7 | 56.6 | 47.0 |
| 36-40h/w | 34.9 | 18.7 | 40.1 | 43.3 | 30.2 | 18.8 | 37.0 | 39.7 | 51.1 | 62.9 | 54.3 | 39.8 |
| Over 40h/w | 36.9 | 66.1 | 29.6 | 19.6 | 42.1 | 65.3 | 32.2 | 14.5 | 58.0 | 62.4 | 58.4 | 34.7 |
| **Burnout complaints** | | | | | | | | | | | | |
| Green | 81.5 | 82.1 | 81.8 | 80.8 | 85.8 | 84.9 | 87.7 | 84.1 | 63.5 | 63.5 | 58.2 | 43.3 |
| Orange | 10.2 | 9.8 | 11.2 | 9.4 | 8.6 | 9.2 | 8.0 | 8.7 | 61.2 | 61.2 | 48.1 | 40.3 |
| Red | 8.3 | 8.1 | 7.0 | 9.8 | 5.5 | 5.9 | 4.3 | 7.2 | 55.1 | 55.1 | 44.5 | 34.9 |

Supplementary Table B: Study measures and Cronbach’s alpha

| **Supplementary Table B: Study Measures and Cronbach's Alpha** | | | | | | | | | | |
| --- | --- | --- | --- | --- | --- | --- | --- | --- | --- | --- |
| *Measurement* | *Item* | *Response alternatives* | *2022* | | | | *2023* | | | |
|  |  |  | *Total* | *Physician* | *Registered nurses* | *Nurse assistants* | *Total* | *Physician* | *Registered nurses* | *Nurse assistants* |
| BAT-12 (Burnout complaints)^a^ | I feel mentally exhausted | 1=Not at all; 2=Rarely; 3=Sometimes; 4=Often; 5=Almost all the time | 0.901 | 0.903 | 0.901 | 0.903 | 0.906 | 0.906 | 0.906 | 0.911 |
|  | I find it hard to recover my energy | 1=Not at all; 2=Rarely; 3=Sometimes; 4=Often; 5=Almost all the time |  |  |  |  |  |  |  |  |
|  | I feel physically exhausted | 1=Not at all; 2=Rarely; 3=Sometimes; 4=Often; 5=Almost all the time |  |  |  |  |  |  |  |  |
|  | I struggle to find any enthusiasm for my work | 1=Not at all; 2=Rarely; 3=Sometimes; 4=Often; 5=Almost all the time |  |  |  |  |  |  |  |  |
|  | I feel a strong aversion towards my job | 1=Not at all; 2=Rarely; 3=Sometimes; 4=Often; 5=Almost all the time |  |  |  |  |  |  |  |  |
|  | I’m cynical about what my work means to others | 1=Not at all; 2=Rarely; 3=Sometimes; 4=Often; 5=Almost all the time |  |  |  |  |  |  |  |  |
|  | I have trouble staying focused | 1=Not at all; 2=Rarely; 3=Sometimes; 4=Often; 5=Almost all the time |  |  |  |  |  |  |  |  |
|  | I have trouble concentrating | 1=Not at all; 2=Rarely; 3=Sometimes; 4=Often; 5=Almost all the time |  |  |  |  |  |  |  |  |
|  | I make mistakes because I have my mind on other things | 1=Not at all; 2=Rarely; 3=Sometimes; 4=Often; 5=Almost all the time |  |  |  |  |  |  |  |  |
|  | I feel unable to control my emotions | 1=Not at all; 2=Rarely; 3=Sometimes; 4=Often; 5=Almost all the time |  |  |  |  |  |  |  |  |
|  | I do not recognize myself in the way I react emotionally | 1=Not at all; 2=Rarely; 3=Sometimes; 4=Often; 5=Almost all the time |  |  |  |  |  |  |  |  |
|  | I may overreact unintentionally | 1=Not at all; 2=Rarely; 3=Sometimes; 4=Often; 5=Almost all the time |  |  |  |  |  |  |  |  |
| Emotional Demands^b^ | Do you have to deal with other people's (not colleagues') personal problems in your work? | 1=Always; 2=Often; 3=Sometimes; 4=Seldom;5= Never/almost never | N/A | N/A | N/A | N/A | N/A | N/A | N/A | N/A |
| Quantitative Demands^b^ | Is your workload unevenly distributed so that work piles up? | 1=Always; 2=Often; 3=Sometimes; 4=Seldom;5= Never/almost never | 0.874 | 0.876 | 0.867 | 0.838 | 0.882 | 0.891 | 0.864 | 0.839 |
|  | How often do you not have time to complete all your tasks? | 1=Always; 2=Often; 3=Sometimes; 4=Seldom;5= Never/almost never |  |  |  |  |  |  |  |  |
|  | Are you falling behind with your work? | 1=Always; 2=Often; 3=Sometimes; 4=Seldom;5= Never/almost never |  |  |  |  |  |  |  |  |
| Illegitimate Work Tasks^c^ | How often do you have to perform tasks that you think ...should be done by someone else? | 1=Very often; 2=Often; 3=Sometimes; 4=Seldom; 5=Never | 0.855 | 0.843 | 0.851 | 0.876 | 0.852 | 0.842 | 0.848 | 0.869 |
|  | ...require more of you than is reasonable? | 1=Very often; 2=Often; 3=Sometimes; 4=Seldom; 5=Never |  |  |  |  |  |  |  |  |
|  | ...put you in unpleasant situations? | 1=Very often; 2=Often; 3=Sometimes; 4=Seldom; 5=Never |  |  |  |  |  |  |  |  |
|  | ...are unfairly assigned to you? | 1=Very often; 2=Often; 3=Sometimes; 4=Seldom; 5=Never |  |  |  |  |  |  |  |  |
|  | How often do you have tasks that you wonder if they ...really need to be done at all? | 1=Very often; 2=Often; 3=Sometimes; 4=Seldom; 5=Never |  |  |  |  |  |  |  |  |
|  | ...sensible and meaningful? | 1=Very often; 2=Often; 3=Sometimes; 4=Seldom; 5=Never |  |  |  |  |  |  |  |  |
|  | ...should exist at all or could be done faster if things were organized differently? | 1=Very often; 2=Often; 3=Sometimes; 4=Seldom; 5=Never |  |  |  |  |  |  |  |  |
|  | ...should exist at all or could be done faster if some others made fewer mistakes? | 1=Very often; 2=Often; 3=Sometimes; 4=Seldom; 5=Never |  |  |  |  |  |  |  |  |
| Effort Reward Imbalance^d^ | I have constant time pressure due to a heavy workload. | 1=Strongly disagree; 2=Disagree; 3=Agree; 4=Strongly agree | 0.784 | 0.758 | 0.786 | 0.832 | 0.779 | 0.747 | 0.776 | 0.829 |
|  | I have many interruptions and disturbances while performing my job. | 1=Strongly disagree; 2=Disagree; 3=Agree; 4=Strongly agree |  |  |  |  |  |  |  |  |
|  | Over the past few years, my job has become more and more demanding. | 1=Strongly disagree; 2=Disagree; 3=Agree; 4=Strongly agree |  |  |  |  |  |  |  |  |
|  | I receive the respect I deserve from my superior or a respective relevant person. | 1=Strongly disagree; 2=Disagree; 3=Agree; 4=Strongly agree | 0.773 | 0.784 | 0.751 | 0.698 | 0.743 | 0.758 | 0.707 | 0.670 |
|  | My job promotion prospects are poor^1^ | 1=Strongly agree; 2=Agree; 3=Disagree; 4=Strongly disagree |  |  |  |  |  |  |  |  |
|  | I have experienced or I expect to experience an undesirable change in my work situation^1^ | 1=Strongly agree; 2=Agree; 3=Disagree; 4=Strongly disagree |  |  |  |  |  |  |  |  |
|  | My job security is poor^1^ | 1=Strongly agree; 2=Agree; 3=Disagree; 4=Strongly disagree |  |  |  |  |  |  |  |  |
|  | Considering all my efforts and achievements, I receive the respect and prestige I deserve at work | 1=Strongly disagree; 2=Disagree; 3=Agree; 4=Strongly agree |  |  |  |  |  |  |  |  |
|  | Considering all my efforts and achievements, my job promotion prospects are adequate. | 1=Strongly disagree; 2=Disagree; 3=Agree; 4=Strongly agree |  |  |  |  |  |  |  |  |
|  | Considering all my efforts and achievements, my salary/income is adequate. | 1=Strongly disagree; 2=Disagree; 3=Agree; 4=Strongly agree |  |  |  |  |  |  |  |  |
| Work-Life Interference^e^ | When I come home from work, I am too tired to do the things I would like to do. | 1=Not at all; 2=Rarely; 3=Sometimes; 4=Often; 5=Almost all the time | 0.929 | 0.934 | 0.927 | 0.925 | 0.926 | 0.928 | 0.926 | 0.924 |
|  | My private life does not look the way I would like it to because of my work. | 1=Not at all; 2=Rarely; 3=Sometimes; 4=Often; 5=Almost all the time |  |  |  |  |  |  |  |  |
|  | I overlook personal problems because of the demands of my work. | 1=Not at all; 2=Rarely; 3=Sometimes; 4=Often; 5=Almost all the time |  |  |  |  |  |  |  |  |
|  | My personal life suffers because of my work. | 1=Not at all; 2=Rarely; 3=Sometimes; 4=Often; 5=Almost all the time |  |  |  |  |  |  |  |  |
|  | I change and adapt my personal life to the demands of the workplace. | 1=Not at all; 2=Rarely; 3=Sometimes; 4=Often; 5=Almost all the time |  |  |  |  |  |  |  |  |
| Control^f^ | In my workplace, I have...enough time with my patients during a typical patient encounter. | 1=To a very high degree; 2=To a fairly high degree; 3=To neither a high nor a low degree; 4=To a fairly low degree; 5=To a very low degree; 6=Not applicable | 0.892 | 0.881 | 0.903 | 0.885 | 0.751 | 0.739 | 0.753 | 0.742 |
|  | ... freedom to make clinical decisions that meet the patient's needs. | 1=To a very high degree; 2=To a fairly high degree; 3=To neither a high nor a low degree; 4=To a fairly low degree; 5=To a very low degree; 6=Not applicable |  |  |  |  |  |  |  |  |
|  | ... the opportunity to provide high-quality care to all patients. | 1=To a very high degree; 2=To a fairly high degree; 3=To neither a high nor a low degree; 4=To a fairly low degree; 5=To a very low degree; 6=Not applicable |  |  |  |  |  |  |  |  |
|  | How much can you influence your working hours based on ... the length of your shifts? | 1=To a very high degree; 2=To a fairly high degree; 3=To neither a high nor a low degree; 4=To a fairly low degree; 5=To a very low degree; 6=Not applicable |  |  |  |  |  |  |  |  |
|  | ... the start of your shifts? | 1=To a very high degree; 2=To a fairly high degree; 3=To neither a high nor a low degree; 4=To a fairly low degree; 5=To a very low degree; 6=Not applicable |  |  |  |  |  |  |  |  |
|  | ... the end of your shifts? | 1=To a very high degree; 2=To a fairly high degree; 3=To neither a high nor a low degree; 4=To a fairly low degree; 5=To a very low degree; 6=Not applicable |  |  |  |  |  |  |  |  |
|  | To what extent do you have control over… which patients you see? | 1=To a very high degree; 2=To a fairly high degree; 3=To neither a high nor a low degree; 4=To a fairly low degree; 5=To a very low degree; 6=Not applicable |  |  |  |  |  |  |  |  |
|  | ... how many patients do you see during the day? | 1=To a very high degree; 2=To a fairly high degree; 3=To neither a high nor a low degree; 4=To a fairly low degree; 5=To a very low degree; 6=Not applicable |  |  |  |  |  |  |  |  |
|  | ... the amount of time for patient appointments? | 1=To a very high degree; 2=To a fairly high degree; 3=To neither a high nor a low degree; 4=To a fairly low degree; 5=To a very low degree; 6=Not applicable |  |  |  |  |  |  |  |  |
|  | ... time for administration or documentation? | 1=To a very high degree; 2=To a fairly high degree; 3=To neither a high nor a low degree; 4=To a fairly low degree; 5=To a very low degree; 6=Not applicable |  |  |  |  |  |  |  |  |
| Social Support^b^ | If you need to, do you get support and help with your work from your first line manager? | 1=Always; 2=Often; 3=Sometimes; 4=Rarely; 5=Never/almost never; 6=Not applicable | N/A | N/A | N/A | N/A | N/A | N/A | N/A | N/A |
|  | If you need to, do you get help and support from your colleagues? | 1=Always; 2=Often; 3=Sometimes; 4=Rarely; 5=Never/almost never; 6=Not applicable | N/A | N/A | N/A | N/A | N/A | N/A | N/A | N/A |
| 1. Reverse coded in analysis | | | | | | | | | | |
| a. English version: Hadžibajramović, E., Schaufeli, W. and De Witte, H. (2020) ‘A Rasch analysis of the Burnout Assessment Tool (BAT)’, *PLOS ONE*. Edited by S. Hoefer, 15(11), p. e0242241.  Swedish version: Hadžibajramović, E. *et al.* (2022) ‘Burnout among midwives—the factorial structure of the burnout assessment tool and an assessment of burnout levels in a Swedish national sample’, *BMC Health Services*  *Research*, 22, p. 1167. | | | | | | | | | | |
| b. English version: Burr, H. *et al.* (2019) ‘The Third Version of the Copenhagen Psychosocial Questionnaire’, *Safety and Health at Work*, 10(4), pp. 482–503.  Swedish version: Berthelsen, H. *et al.* (2020) ‘Validation of the Copenhagen Psychosocial Questionnaire Version III and Establishment of Benchmarks for Psychosocial Risk Management in Sweden’, *International Journal of*  *Environmental Research and Public Health*, 17(9), p. 3179. | | | | | | | | | | |
| c. English version: Jacobshagen, N. (2006) *Illegitimate tasks, illegitimate stressors: Testing a New stressor-strain concept*. Bern: University of Bern.  Swedish version: Stengård, J., Leineweber, C. and Berthelsen, H. (2024) ‘Illegitimate work tasks: an investigation of psychometric properties of the Swedish version of the BITS instrument and its suitability in human versus “non-  human” service occupations’, *BMC Public Health*, 24(1), p. 1935. | | | | | | | | | | |
| d. English version: Siegrist, J. *et al.* (2004) ‘The Measurement of Effort-Reward Imbalance at Work’, *Social Science & Medicine*, (58), pp. 1483–1499.  Swedish version: Leineweber, C. *et al.* (2010) ‘How valid is a short measure of effort–reward imbalance at work? A replication study from Sweden’, *Occupational and Environmental Medicine*, 67(8), pp. 526–531. | | | | | | | | | | |
| e. English version: Fisher, G.G., Bulger, C.A. and Smith, C.S. (2009) ‘Beyond work and family: A measure of work/nonwork interference and enhancement.’, *Journal of Occupational Health Psychology*, 14(4), pp. 441–456.  Swedish version: Magnusson Hanson, L.L. *et al.* (2018) ‘Cohort Profile: The Swedish Longitudinal Occupational Survey of Health (SLOSH)’, *International Journal of Epidemiology*, 47(3), pp. 691–692i. | | | | | | | | | | |
| f. Swedish version: Albrecht, S.C. *et al.* (2017) ‘The longitudinal relationship between control over working hours and depressive symptoms: Results from SLOSH, a population-based cohort study’, *Journal of Affective Disorders*, 215,  pp. 143–151.; and Magnusson Hanson, L.L. *et al.* (2018) ‘Cohort Profile: The Swedish Longitudinal Occupational Survey of Health (SLOSH)’, *International Journal of Epidemiology*, 47(3), pp. 691–692i. | | | | | | | | | | |

Supplementary Table C: Mean values and correlation

Mean values for burnout complaints each year and baseline job demands and job resources and correlation between variables

| **Supplementary Table C: Mean values for burnout complaints each year and baseline job demands and job resources and correlation between variables** | | | | | | | | | | | | | | | |
| --- | --- | --- | --- | --- | --- | --- | --- | --- | --- | --- | --- | --- | --- | --- | --- |
|  |  | *Mean values* | | | | *Pearson Correlation* | | | | | | | | | |
|  |  | *Physicians* | *Registered. Nurses* | *Nurse Assistants* | *Total* | *1* | *2* | *3* | *4* | *5* | *6* | *7* | *8* | *9* | *10* |
| **Burnout complaints 2022 ^a^** | *n* | 1613 | 1595 | 818 | 4026 | 1 |  |  |  |  |  |  |  |  |  |
|  | Mean (95% CI) | 1.91(1.88;1.94) | 1.81(1.78;1.84) | 1.89(1.85;1.93) | 1.86(1.84;1.88) |  |  |  |  |  |  |  |  |  |  |
| **Burnout complaints 2023 ^a^** | *n* | 1626 | 1584 | 806 | 4016 | 0.681** | 1 |  |  |  |  |  |  |  |  |
|  | Mean (95% CI) | 1.86(1.83;1.89) | 1.76(1.73;1.79) | 1.87(1.82;1.91) | 1.82(1.80;1.84) |  |  |  |  |  |  |  |  |  |  |
| **Emotional Demands ^b^** | n | 1643 | 1626 | 845 | 4114 | 0.181** | 0.134** | 1 |  |  |  |  |  |  |  |
|  | Mean (95% CI) | 3.36(3.30;3.42) | 3.40(3.35;3.46) | 3.19(3.11;3.26) | 3.34(3.31;3.38) |  |  |  |  |  |  |  |  |  |  |
| **Quantitative Demands ^b^** | *n* | 1642 | 1622 | 842 | 4106 | 0.460** | 0.346** | 0.245** | 1 |  |  |  |  |  |  |
|  | Mean (95% CI) | 3.24(3.19;3.28) | 2.81(2.77;2.86) | 2.62(2.56;2.68) | 2.94(2.91;2.97) |  |  |  |  |  |  |  |  |  |  |
| **Illegitimate Work Tasks ^c^** | *n* | 1629 | 1606 | 832 | 4067 | 0.502** | 0.402** | 0.282** | 0.494** | 1 |  |  |  |  |  |
|  | Mean (95% CI) | 3.08(3.05;3.12) | 2.91(2.87;2.94) | 2.87(2.81;2.92) | 2.97(2.95;2.99) |  |  |  |  |  |  |  |  |  |  |
| **Effort Reward Imbalance ^d^** | *n* | 1611 | 1592 | 829 | 4032 | 0.536** | 0.389** | 0.227** | 0.451** | 0.572** | 1 |  |  |  |  |
|  | Mean (95% CI) | 1.23(1.20;1.25) | 1.35(1.32;1.37) | 1.45(1.40;1.49) | 1.32(1.30;1.34) |  |  |  |  |  |  |  |  |  |  |
| **Work-Life Interference ^e^** | *n* | 1638 | 1619 | 836 | 4093 | 0.662** | 0.511** | 0.226** | 0.491** | 0.487** | 0.572** | 1 |  |  |  |
|  | Mean (95% CI) | 3.15(3.10;3.20) | 3.02(2.96;3.07) | 3.04(2.96;3.11) | 3.07(3.04;3.10) |  |  |  |  |  |  |  |  |  |  |
| **Control ^f^** | *n* | 1608 | 1584 | 777 | 3969 | -0.371** | -0.286** | -0.093** | -0.238** | -0.378** | -0.477** | -0.419** | 1 |  |  |
|  | Mean (95% CI) | 2.77(2.73;2.81) | 2.87(2.82;2.91) | 2.48(2.42;2.54) | 2.75(2.72;2.78) |  |  |  |  |  |  |  |  |  |  |
| **Managerial Support ^g^** | *n* | 1569 | 1577 | 820 | 3966 | -0.342** | -0.234** | -0.126** | -0.245** | -0.344** | -0.467** | -0.307** | 0.315** | 1 |  |
|  | Mean (95% CI) | 3.68(3.62;3.73) | 3.58(3.52;3.64) | 3.55(3.47;3.64) | 3.61(3.58;3.65) |  |  |  |  |  |  |  |  |  |  |
| **Collegial Support ^g^** | *n* | 1634 | 1618 | 846 | 4098 | -0.295** | -0.205** | -0.018 | -0.200** | -0.241** | -0.221** | -0.245** | 0.197** | 0.349** | 1 |
|  | Mean (95% CI) | 4.34(4.30;4.38) | 4.45(4.42;4.48) | 4.30(4.25;4.35) | 4.38(4.35;4.40) |  |  |  |  |  |  |  |  |  |  |
| a) Scale 1-5, (1) "Not at all" to (5) "Almost all the time" | | | | | | | | | | | | | | | |
| b) Scale 1-5, (1) "Always" to (5) “Never/Almost never”. | | | | | | | | | | | | | | | |
| c) Scale 1-5, (1)” Very often” to (5) “Never” | | | | | | | | | | | | | | | |
| d) Effort Reward Imbalance Ratio 0-4; Effort Reward Imbalance items scale 1-4, (1) “Strongly agree” to (4) “Strongly disagree” | | | | | | | | | | | | | | | |
| e) Scale 1-5, (1) “Not at all” to (5) “Almost all the time” | | | | | | | | | | | | | | | |
| f) Scale 1-5, (1) “To a very high degree” to (5) “To a very low degree” | | | | | | | | | | | | | | | |
| g) Scale 1-5, (1) “Always” to (5) “Never/Almost never” | | | | | | | | | | | | | | | |

Supplementary: Plots for Moderation

Note: Filled line = linear association between job demands and subsequent burnout complaints without moderation; the y-axis is broken

**Supplementary figures for Table 3 - Entire Sample - Statistically Significant Moderation**

*Moderation by control*


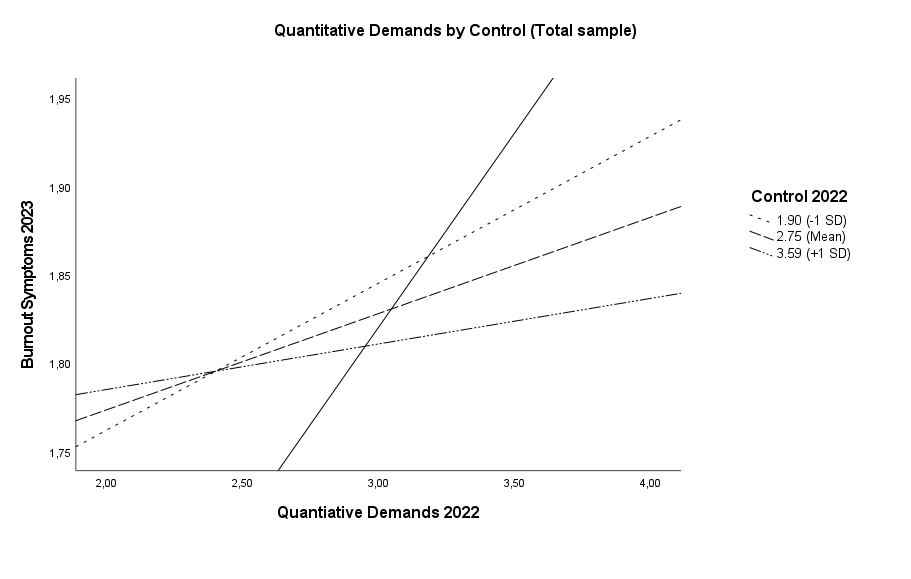
Figure A


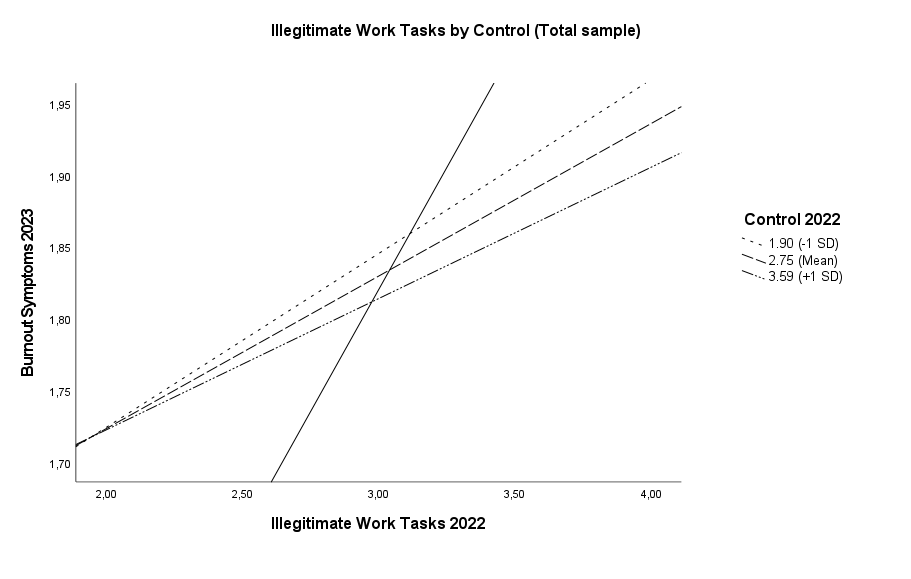
Figure B


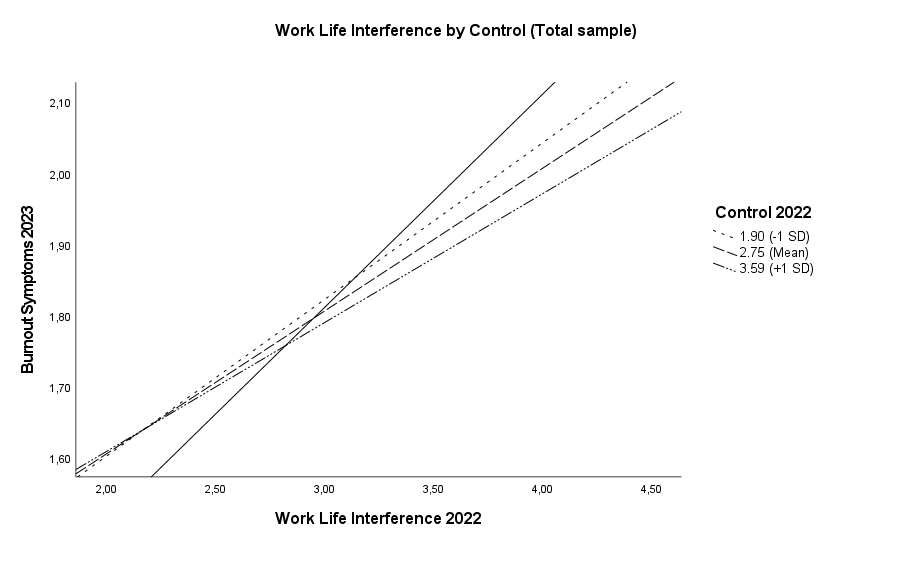
Figure C

**Supplementary figures for Table 3 – Each Profession - Statistically Significant Moderation**

Note: Filled line = linear association between job demands and subsequent burnout complaints without moderation; the y-axis is broken

Physicians: Moderation by control


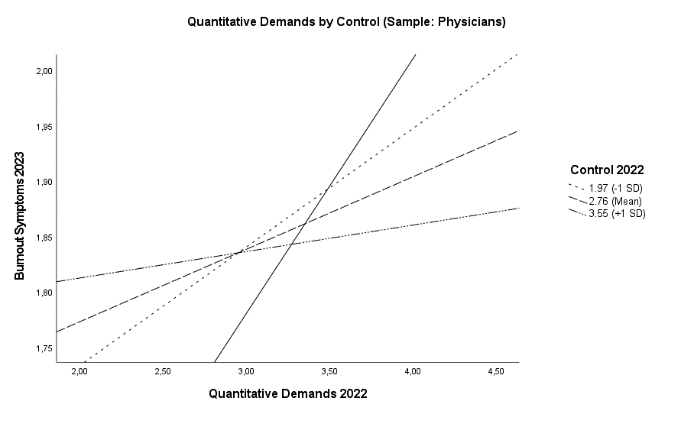
Figure D


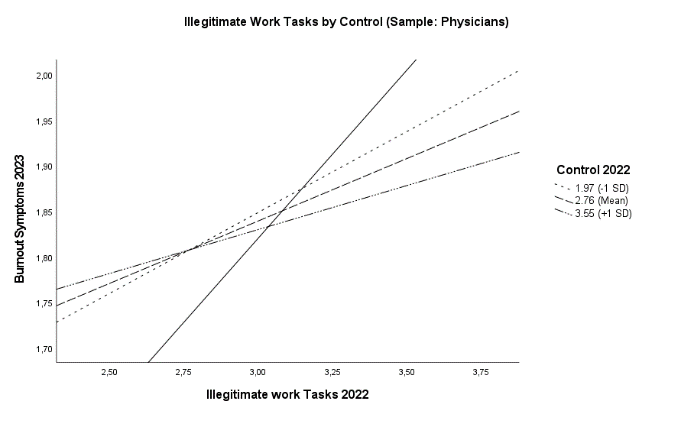
Figure E


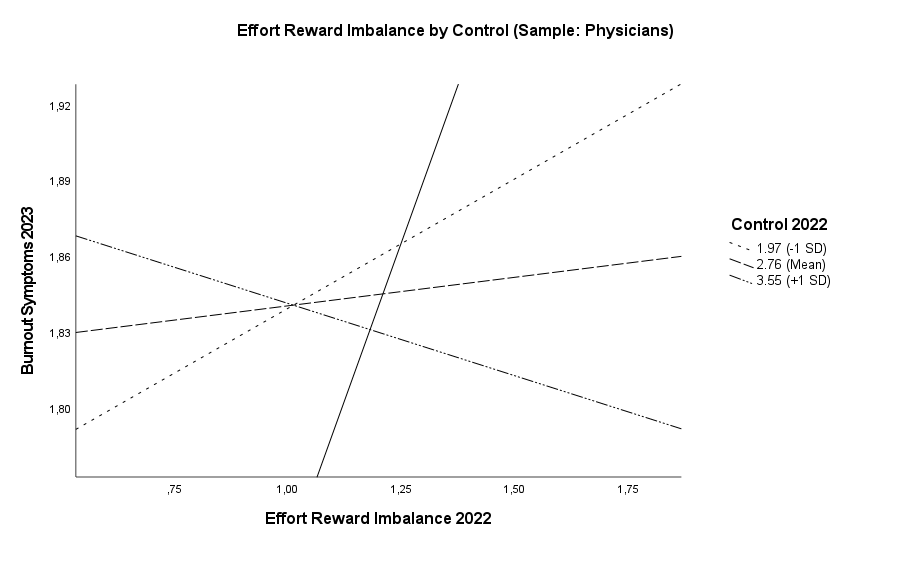
Figure F

Registered Nurses: Moderation by collegial support


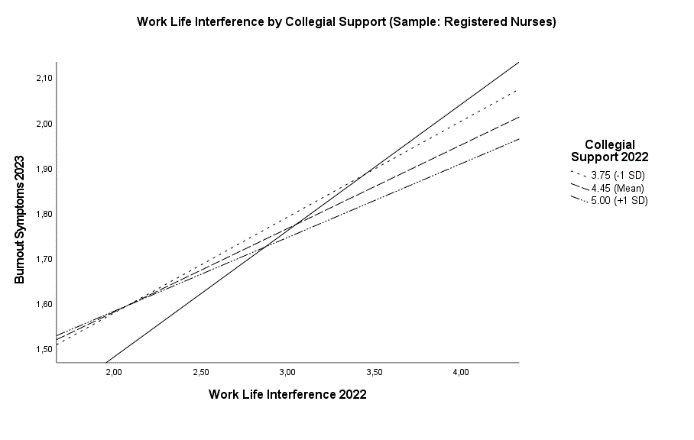
Figure G

**Supplementary figures for Table 4 - Statistically Significant Moderation**

*Moderation by Control and Profession*

Figure H

**
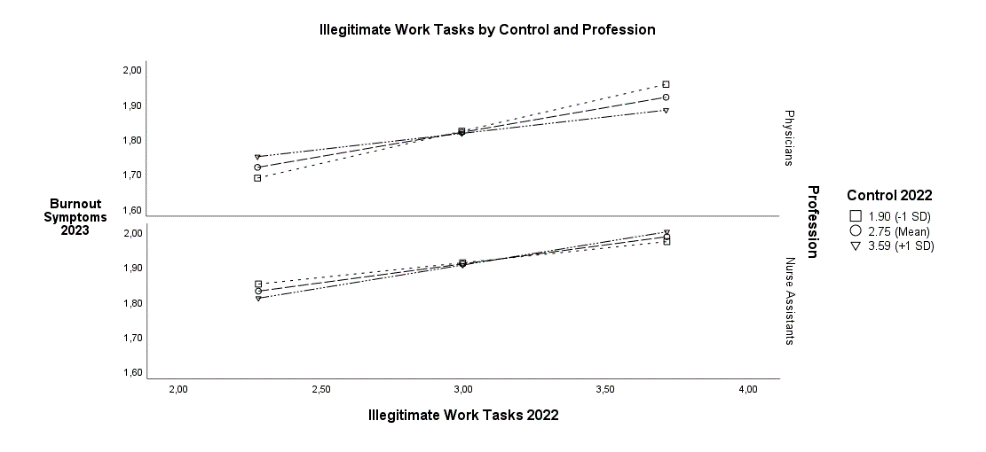
**

Figure I

**
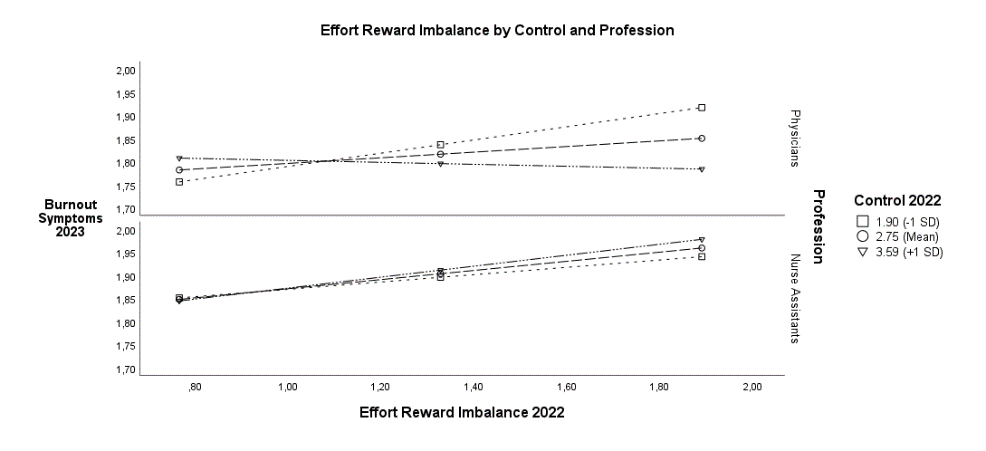
**
